# Profile of motorcycle trauma in a referral hospital in Northeast Brazil

**DOI:** 10.1590/1806-9282.20260639

**Published:** 2026-10-30

**Authors:** Thábata Gois, Ariano Oliveira, Vinicius Bacurau, Francisco Dantas, Luana Dutra, Laura Leite, Daniel Oliveira

**Affiliations:** 1 Universidade Potiguar – Natal (RN), Brazil.; 2 Hospital Monsenhor Walfredo Gurgel, Department of General Surgery – Natal (RN), Brazil.; 3 Universidade Potiguar, Department of Surgery – Natal (RN), Brazil.

**Keywords:** Accidents, traffic, Emergency service, hospital, Traumatology, Multiple trauma, Epidemiology

## Abstract

**OBJECTIVE:**

The aim of this study was to analyze the profile of motorcycle accident victims treated at Hospital Monsenhor Walfredo Gurgel, a trauma referral center in Rio Grande do Norte, between February and October 2024.

**METHODS:**

This was an observational, retrospective, and descriptive study. Data regarding epidemiological profiles, mode of arrival, medical specialties involved, length of hospital stay, and clinical outcomes were extracted from electronic medical records.

**RESULTS:**

Among 7,176 recorded victims, there was a clear male predominance (75.52%, n=5,420), while females accounted for 24.47% (n=1,756). Analyzing victims aged 21–70 years, most cases occurred in the 21–30 age group (40.67%), followed by the 31–40 age group (28.66%). Most patients (71.27%) were admitted using specialized transport, including rural and state SAMU ambulances. General Surgery was the specialty most frequently involved in care (52.89%, n=3,178), followed by Orthopedics and Traumatology (36.98%), with Neurosurgery, Oral and Maxillofacial Surgery, Neurology, and Urology accounting for smaller proportions.

**CONCLUSION:**

Motorcycle injuries predominantly affect young, economically active men aged 21–40 years, accentuating the need for preventive strategies to reduce the burden on health services and improve pre-hospital and in-hospital care.

## INTRODUCTION

Traffic accidents represent a serious public health problem in Brazil, with significant social and economic impacts. Among the various types of accidents, those involving motorcycles stand out due to the high incidence of injuries and deaths, especially among young people. In 2024, the Northeast region concentrated nearly a third of all national hospitalizations resulting from motorcycle trauma in Brazil, recording 54,566 of the 165,431 overall cases (32.98%)^1^.

Beyond their public health burden, traffic accidents impose a substantial economic strain, with annual national costs estimated at approximately R$ 50 billion due to healthcare expenses and lost economic productivity. This financial impact scales directly with injury severity, as fatal outcomes generate disproportionately higher socioeconomic losses through workforce depletion^2^.

Motorcyclist mortality represents a critical public health concern, with 13,340 deaths recorded nationwide in 2023 among individuals aged 15 years and older, peak mortality occurring in the 20–29 age bracket, and the Northeast region accounting for the highest proportion of fatalities (5,294 deaths; 39.68%)^2^. Since this demographic overlaps heavily with the economically active population, premature mortality in young adults directly compromises workforce availability, escalating public healthcare expenditures and generating long-term socioeconomic losses^2,3^.

From a clinical perspective, the classic trimodal distribution of trauma mortality characterizes death timing into three distinct phases: immediate deaths occurring within minutes due to non-survivable injuries, early deaths within hours, where survival hinges directly on rapid prehospital triage and emergency care during the "golden hour," and late deaths occurring days to weeks post-injury from hospital complications^4^. Aligning with the United Nations Sustainable Development Goal to halve global road traffic fatalities^5^, understanding regional epidemiological profiles becomes imperative. Therefore, this study aimed to characterize motorcycle trauma victims at a referral center in Northeast Brazil, evaluating transport modes, length of stay, and clinical outcomes to inform preventive traffic policies and optimize hospital resource allocation.

## METHODS

This is a retrospective, observational, descriptive study. Data were collected by the researchers from the internal software of Hospital Monsenhor Walfredo Gurgel (HMWG), a trauma referral hospital in Rio Grande do Norte, Brazil, with support from the hospital's data management and statistics team, under the supervision of the study coordinator.

Patients aged 21–70 years with motorcycle-related trauma admitted to HMWG between February 1, 2024, and October 31, 2024, were included. This age restriction reflected the age categorization available in the hospital information system. Records involving non-motorcycle-related trauma or lacking sufficient information to confirm the mechanism of injury were excluded.

The variables analyzed included demographic, clinical, and healthcare-related characteristics. Demographic and epidemiological variables included age at hospital admission, sex (male or female), and patients’ origin, categorized as the capital city or inland municipalities of Rio Grande do Norte. Healthcare-related variables included the mode of arrival at the hospital, categorized according to the type of transportation used: emergency medical services (SAMU), ambulance transfer, or private transportation. Clinical variables included the medical specialties involved in patient care, length of hospital stay in days, and clinical outcomes such as hospital discharge, transfer to another healthcare facility, death, discharge against medical advice, discharge with planned return, and unavailable/inconclusive records.

Data were organized and analyzed by the researchers using descriptive statistics. Absolute and relative frequencies were calculated for the analyzed variables to describe the epidemiological, clinical, and healthcare-related profile of patients with motorcycle-related trauma.

The study was approved by the Research Ethics Committee (CEP) of Universidade Potiguar (UnP), under protocol number 7.602.158, on May 28, 2025, and conducted in accordance with the ethical principles of the Declaration of Helsinki. As this was a retrospective study based on medical record review, there was no direct contact between researchers and patients, and the clinical management during hospitalization was not influenced by the study procedures.

## RESULTS

From February to October 2024, 7,176 patients who were victims of motorcycle-related trauma were treated. Predominantly male patients were observed, with 5,420 (75.5%) cases, while females accounted for 1,756 (24.5%). Among these, the profile of patients aged 21–70 years (n=6,008) was analyzed, with the majority of cases concentrated in the 21–30 age group (2,444; 40.3%), followed by the 31–40 age group (1,722; 28.4%), 41–50 years (1,114; 18.4%), 51–60 years (530; 7.3%), and 61–70 years (198; 2.7%) (Table 1). The remaining age groups (0–20 and 71 years or older) represent 16%.

**Table 1:** Sample variables (n=6,008).

Category		n	Percentage (%)
Age group			
	21–30	2,444	40.67
	31–40	1,722	28.66
	41–50	1,114	18.54
	51–60	530	8.82
	61–70	198	3.29
Mode of arrival			
	Rural area ambulance	1,921	31.97
	Natal SAMU ambulance	1,409	23.45
	State SAMU ambulance	952	15.84
	Walk-in patients	966	16.07
	Other sources	760	12.64
Specialty			
	General surgery	3,178	52.89
	Orthopedics and traumatology	2,222	36.98
	Neurosurgery	343	5.70
	Other medical specialties	265	4.41

SAMU: Mobile Emergency Care Service.

The monthly number of cases in the selected age group ranged from 575 to 743 throughout the study period, with a mean of 667.6 cases per month (SD 61.4). Higher monthly counts were observed between July and October, peaking in August (743 cases), followed by September (730), July (726), and October (717). During the remaining months, the number of cases ranged from 575 to 642.

Most patients were admitted using specialized transport, with the primary sources being: ambulances from rural areas (1,921; 31.97%), Natal SAMU ambulances (1,409; 23.45%), and state SAMU ambulances (952; 15.84%). Walk-in patients accounted for 16.07% of attendances (966), while other sources, such as emergency care units (UPAs), the Fire Department, municipal and private hospitals, added up to 12.64% (Table 1).

The specialty most frequently involved in patient care was General Surgery, accounting for 3,178 cases (52.89%). Next were Orthopedics and Traumatology (2,222; 36.98%) and Neurosurgery (343; 5.70%). Other specialties, such as Oral and Maxillofacial Surgery, Neurology, and Urology, were involved in smaller proportions (Table 1).

In total, 1,902 patients required hospitalization (31.65% of the study group). The monthly distribution of hospitalizations ranged from 177 to 231 cases, with a mean value of 211.3 (SD 15.7). The average length of hospital stay showed a downward trend throughout the year, decreasing from 7.09 days in February to 4.1 days in October (mean value 5.82, and SD 0.90).

Most hospitalized patients were discharged. Among these, 952 (50.05%) had clinical improvement, and 99 (5.20%) were discharged with complete resolution of their condition. A total of 663 (34.85%) were transferred to other healthcare facilities. There were 33 deaths (1.7%), of which 17 were certified by the Forensic Medic Institute, 13 by the attending physician, and 3 identified through analysis by the Death Investigation Service. Other less frequent outcomes—such as hospital discharge against medical advice, discharge with expected return, and inconclusive data—totaled 155 patients (7.88%).

## DISCUSSION

In the present study, motorcycle trauma predominantly affected young adult males aged 21–40 years, aligning with regional and international literature demonstrating marked male vulnerability to traumatic events^6^. This trend is largely attributed to the "young male syndrome," a behavioral paradigm wherein young men exhibit a higher propensity for risk-taking, often driven by evolutionary intrasexual competition and social status seeking^7^. Moreover, male predominance extends across broader age groups, with traffic collisions, falls, and interpersonal violence consistently reported as primary etiologies for severe injuries, including traumatic brain injury^8^. Consequently, implementing targeted public health interventions for high-risk male demographics remains essential to mitigate these preventable outcomes.

Notably, the highest incidence of injuries occurred among individuals aged 21–30 years (40.3%), a demographic cohort that constitutes a vital core of the economically active population^4^. Premature morbidity and disability in this productive age group severely deplete the workforce, escalating public healthcare expenditures, straining social security systems, and placing substantial financial and caregiving burdens on affected families^4,5^.

Regarding in-hospital management, general surgery represented the highest demand for specialized consultations, followed by orthopedics and neurosurgery. Although previous literature reports a primary predominance of orthopedic and neurosurgical admissions, driven by the high frequency of limb, head, and neck injuries in motorcycle collisions^6^, the referral hospital evaluated in this study exhibited a higher volume of general surgical interventions, reflecting distinct regional trauma triage patterns and initial stabilization requirements.

When analyzing the temporal distribution of traffic incidents, a higher volume of hospital admissions among motorcyclists was observed between July and October. While the limited observational period precludes establishing a definitive seasonal pattern, this temporal cluster aligns with national road safety reporting that demonstrates a progressive rise in traffic infractions throughout the year^9^. Rather than proving direct causality, this overlap highlights the utility of tracking short-term temporal trends to guide seasonal resource allocation and targeted enforcement strategies.

Concerning pre-hospital care, most patients were admitted via specialized transport, including ambulance transfers from rural municipalities and state pre-hospital networks. This finding demonstrates a high reliance on regional referral pathways for trauma management in the state. Although transfer times were not directly measured in this study, the high proportion of patients arriving via inter-facility transport suggests potential challenges related to geographical distances, reinforcing the importance of optimizing pre-hospital triage and transport logistics to mitigate delays in definitive trauma care^4^.

In terms of length of stay, the average hospitalization duration in the evaluated period was 5.8 days. Compared with aggregate public health registry averages, such as the state mean for Rio Grande do Norte (6.8 days) and higher regional extremes nationwide reaching 13.7 days^1^, the institutional metrics demonstrate favorable bed turnover and operational resolution. While direct cross-system comparisons require caution due to differences in case-mix severity, this shorter hospital stay suggests efficient clinical bed management in processing acute trauma admissions.

On a national scale, population-based epidemiological surveillance confirms a heavy burden of transport-related mortality, with motorcycle crashes driving a high volume of hospital admissions nationwide^10^ and contributing to nearly a third of all traffic fatalities^11^. In the present study, the in-hospital mortality rate was 1.7%. Although population-level mortality and tertiary in-hospital outcomes represent distinct methodological frameworks, this relatively low inpatient fatality rate suggests that, despite the high mortality associated with traffic accidents nationwide, the cases treated at this hospital had relatively low hospital mortality, possibly reflecting the characteristics of the patients and the quality of care provided. However, it is important to note that 663 patients (34.8%) were transferred to other healthcare facilities, resulting in loss to follow-up. Consequently, the true overall mortality may be underestimated, as post-transfer clinical outcomes and late fatalities could not be tracked.

Certain methodological limitations warrant consideration when interpreting these findings. First, the retrospective design and reliance on routinely collected medical records limited the availability of certain clinical variables. Second, conducted at a single tertiary referral center, the findings primarily reflect the characteristics of patients treated within this specific healthcare setting and may not fully represent the broader population of motorcycle-related trauma patients, particularly those with less severe injuries managed at other facilities. Finally, the rate of inter-hospital transfers introduced loss to follow-up, precluding the assessment of post-discharge mortality and late complications. Despite these constraints, this study provides a robust epidemiological and operational profile of motorcycle trauma at the main referral trauma hospital in Rio Grande do Norte. By delivering real-world data, these findings offer actionable insights for public traffic safety policies, prehospital resource optimization, and regional trauma network planning, establishing a foundational framework for future prospective multicenter investigations.

## CONCLUSION

The epidemiological profile of trauma victims, resulting from motorcycle accidents treated at a tertiary referral trauma center, revealed a predominance of young adult males within their prime economically active years. The high reliance on inter-hospital transfers from rural interior regions emphasizes ongoing regional logistics challenges in prehospital stabilization and timely transfer to definitive care. Analysis of length of stay and clinical outcomes showed a relatively short average hospital stay and low in-hospital mortality, suggesting adequate service capacity, although limited by incomplete follow-up of patients transferred to other facilities. The predominance of cases involving General Surgery, Orthopedics and Traumatology, and Neurosurgery also contributes to more efficient planning for the allocation of human and structural resources in trauma services. Therefore, the findings of this study have broadened our understanding of the epidemiological overview of motorcycle accidents in the region and may provide relevant insights for the development of public policies aimed at accident prevention, strengthening traffic education and enforcement, and decentralizing the prehospital care network.

## Data Availability

The datasets generated and/or analyzed during the current study are available from the corresponding author upon reasonable request.
